# Further Experience with Vaccine in Tuberculosis

**Published:** 1910-06

**Authors:** E. C. Thrash

**Affiliations:** Atlanta, Ga.; Prof. Bacteriology and Pathology, and Lecturer on Tuberculosis Atlanta School of Medicine; Visiting Physician on Chest Diseases to Tabernacle Infirmary, Physician to Clinic of Atlanta Tuberculosis Society, Etc.


### FURTHER EXPERIENCE WITH VACCINE IN TUBERCULOSIS.

BY DR. E. C. THRASH, ATLANTA, GA.

*Prof. Bacteriology and Pathology, and Lecturer on Tuberculosis Atlanta School of Medicine; Visiting Physician on Chest Diseases to Tabernacle Infirmary, Physician to Clinic of Atlanta Tuberculosis Society, Etc.*

There are a few thoughts I wish to present as a foreword to this essay that have been prompted from my handling tuberculosis in a general way. No disease is attracting so much attention at present from the world as tuberculosis, and justly so, but there are many grave errors being made by well meaning, but untrained, phthisiphobic people, and it is the duty of the medical profession to attempt to correct them. The warfare against this disease is being fiercely waged, but it is accompanied with too much glamour, sounding of trumpets, ignorant exploitations, and frightening, panic-producing vaporings from those utterly devoid of a proper knowledge of the subject. The medical profession is the proper source from which this work should come, and it should be done quietly, with an eye always to avoiding panic-stricken conditions as they now exist. We have reached a point already where we have as difficult a problem on hand in curing the public of phthisiphobia as of ridding it of tuberculosis. What it needs to be taught is truth, and we who are giving our energies, our studies, and even our lives to this work feel keenly our incapacity in properly presenting it. This being so, then what should we expect but to produce a wild delirium of fright and consternation, putting the people to running to they know not whither and from they know not what, when we are so foolhardy as to call in the clergy, the club-women, teachers, philanthropists, etc., entirely without proper technical and medical training along this line to help to alleviate the world from this scourge.

In my tuberculosis work for the past few years, I have attempted to make painstaking observations as to the value of vaccine, both as diagnostic annd therapeutic agents. It shall be the object of this paper, not to enter into the minute details of their administration, but to give the general conclusions I have reached from observation in their use. In summarizing these, I wish to state emphatically that he who depends upon tuberculin alone either for diagnosis or treatment, will be sorely disappointed, but on the other hand, taken with everything else that is available in the proper handling of tuberculosis, I consider no agent so valuable as this for, frequently after exhausting every other means to make a diagnosis, being still in doubt, tuberculin will often turn the balance and prove an invaluable aid in clearing the doubt. No one who is not a good clinician and diagnostician will get satisfactory results from vaccine. The same good judgment is needed in interpreting the results from the use of these, as is needed in the interpretation of one's clinical findings. Four of these tests are worthy of mentioning—the sub-cuticular, Von Pirquet's cuticular, Moro's ointment, and the systemic. They are mentioned in the order of their delicacy, but not in that of their administration, as the sub-cuticular will sometimes give a systemic reaction and interfere with the local reaction, should one wish to administer the others later. Von Pirquet's and Moro's can be used together, and if these do not clear the doubt the other two may then be used, but separately, using the sub-cuticular first. Let me say this, any person who is sick enough to go to a physician for an examination thinking there is a possibility of his having tuberculosis deserves the most painstaking examination and the utilization of every thing one has at hand to enable him to arrive at the truth—spending not a few minutes, but hours, days or weeks until he can say yea or nay with some degree of positiveness.

It is superfluous to say that tuberculin is beneficial and curative in the treatment of tuberculosis. This has been and is the decision of practically everyone who has used it extensively, carefully, wisely, and prudently. The longer and the more one uses it, the more profoundly impressed he becomes with its potency for both good and evil, and the more he insists upon its careful administration. But because it must be used properly to get results, and will produce harm used improperly, would no more preclude its use than the same thing would preclude the administration of strychnine or digitalis. There is great need of a more accurate standardization of tuberculin. On account of difference in virulence of different strains of organisms it varies in antigen potency, in proportion as the different strains from which it is made vary. So a mg. of a tuberculin made from one strain of organisms would be practically sure to vary in antigen potency from a mg. of one from another strain. This difficulty with our present knowledge is impossible to overcome, but there is one in reference to a more uniform nomenclature which can be, and that is the variable strength of the solutions made by different manufacturers, and the references by one to the dilution, and to another to tubercular solids when speaking of doses. To be explicit, T. R. *solution* carries 10 mg of solids to the cc.; and B. E. put up by some manufacturers carries 5 mg. to the c. c., while others put one mg. of solids to the c. c. It makes quite a great difference as to whether one refers to solids or solutions in reference to doses. We should have a universal plan, in my opinion, of referring to solids when we name the dose, and in this way much confusion could be avoided. In the paper read before the association one year ago, I referred to solutions when doses were mentioned, I have begun since that time to estimate my doses exclusively in solids. This makes a difference of from 200 to 1,000 fold less, owing to the dilution, in each dose. That is, .001 of a mg. of the solution would be equal to .000001 mg. of solids, where the solution is 1 mg. per c. c., or 000005 when it is 5 mg. per c. c. This method applies only to B. E. and T. R., as the other forms of tuberculin are filtrates, and we can only estimate the dose in each respective solution according as it has been standardized by its originator.

I am now using only B. E. in my therapeutic work, though I am not prepared to say whether or not it is better than T. R. We should unquestionably select one or the other of these as the best of them all as munizing agents. The dosage should be from .0000001 to .000001 of a mg. of solids. Even doses so small as these will often give most marked reaction. These doses are repeated from one to three times a week, owing to the progress the patient makes, and the degree to which he tolerates the vaccine. I increase my dosage much more slowly than formerly, making an increase only once in every two or three weeks, and then not over 10 per cent. Covering a period of one year, the quantities and length of time between administration will run a ragged, irregular up and down course with a tendency upward in dosage and lengthening the time between administering them, reaching a maximum of .001 mg. and giving a dose every two or three weeks. I rarely give over .001 mg. except as a test dose, and for this I rarely give over .01 mg. as a dose. Since reporting my work to the Association last April I have had under treatment in my private practice 56 cases to whom tuberculin has been administered. I make no mention of the cases where vaccines have not been given, as they are principally third stage cases that have the usual line of treatment and are uneventful. Neither do I mention the cases treated under the auspices of the Anti-Tubercular Association of Atlanta, many of whom I am treating with vaccines, as these like all outdoor clinical patients cannot be handled in a way to make one's observations of much practical value. The cases to which I refer embrace only those who have been treated as long as two months, as the ones treated for a shorter time could not possibly make much change. I shall not read the tabulated list of these cases at length, but will only give a summary, which is as follows:

First stage.—Number treated, 16; apparently well, 10; improved, 5; not improved, 1.

Second stage.—Number treated, 14; apparently well, 5; arrested, 2; improved, 6; not improved, 1.

Third stage.—Number treated, 26; arrested, 3; improved, 10; not improved, 13.

Following is a more extended report:

It is only those cases that are more or less chronic that have responded well to treatment at my hands. A few of the third stage chronic cases have responded well. Many of the second stage cases and all of the first stage ones except those whose resistance is almost nil, the disease acute and progress rapid. I wish to state in conclusion that tuberculin therapeutics is rational and scientific in theory and that experience has demonstrated its practical value. Its administration has been such that it not only warrants my continuing its use, but assures me that it is par excellence, the best treatment in properly selected cases of tuberculosis.

